# Mesenchymal to amoeboid transition is associated with stem-like features of melanoma cells

**DOI:** 10.1186/1478-811X-12-24

**Published:** 2014-04-01

**Authors:** Maria Letizia Taddei, Elisa Giannoni, Andrea Morandi, Luigi Ippolito, Matteo Ramazzotti, Maurizio Callari, Paolo Gandellini, Paola Chiarugi

**Affiliations:** 1 Department of Experimental and Clinical Biomedical Sciences, Tuscany Tumor Institute, University of Florence, viale Morgagni 50, Florence 50134, Italy; 2 Department of Experimental Oncology and Molecular Medicine, Fondazione IRCCS Istituto Nazionale dei Tumori, Milan 20133, Italy; 3 Center for Research, Transfer and High Education ‘Study at Molecular and Clinical Level of Chronic, Inflammatory, Degenerative and Neoplastic Disorders for the Development on Novel Therapies’, Florence, Italy

**Keywords:** Melanoma, Cell plasticity, Amoeboid motility, Stemness, EphA2

## Abstract

**Background:**

Cellular plasticity confers cancer cells the ability to adapt to microenvironmental changes, a fundamental requirement for tumour progression and metastasis. The epithelial to mesenchymal transition (EMT) is a transcriptional programme associated with increased cell motility and stemness. Besides EMT, the mesenchymal to amoeboid transition (MAT) has been described during tumour progression but to date, little is known about its transcriptional control and involvement in stemness. The aim of this manuscript is to investigate (i) the transcriptional profile associated with the MAT programme and (ii) to study whether MAT acquisition in melanoma cancer cells correlates with clonogenic potential to promote tumour growth.

**Results:**

By using a multidisciplinary approach, we identified four different treatments able to induce MAT in melanoma cells: EphA2 overexpression, Rac1 functional inhibition using its RacN17 dominant negative mutant, stimulation with Ilomastat or treatment with the RhoA activator Calpeptin. First, gene expression profiling identified the transcriptional pathways associated with MAT, independently of the stimulus that induces the MAT programme. Notably, gene sets associated with the repression of mesenchymal traits, decrease in the secretion of extracellular matrix components as well as increase of cellular stemness positively correlate with MAT. Second, the link between MAT and stemness has been investigated *in vitro* by analysing stemness markers and clonogenic potential of melanoma cells undergoing MAT. Finally, the link between MAT inducing treatments and tumour initiating capability has been validated *in vivo*.

**Conclusion:**

Taken together, our results demonstrate that MAT programme in melanoma is characterised by increased stemness and clonogenic features of cancer cells, thus sustaining tumour progression. Furthermore, these data suggest that stemness is not an exclusive feature of cells undergoing EMT, but more generally is associated with an increase in cellular plasticity of cancer cells.

## Background

Plasticity in cell motility is a key prerequisite for the metastatic dissemination of tumour cells. Indeed, cancer cells may achieve different types of cell motility, including mesenchymal, collective, and amoeboid styles. Thus, by shifting between different motility styles, migrating cells can adapt to environmental changes and matrix stiffness to elude anticancer treatments, which represents a major challenge for developing strategies aimed at blocking the dissemination of cancer cells [1-3]. Mesenchymal motility depends on extra-cellular matrix (ECM) proteolysis through production of matrix metalloproteinases (MMPs). It is characterised by activation of Rac1 at the leading edge of the cell, and inhibition of RhoA GTPase [3], conferring to migrating cells an elongated and polarized cell morphology. Conversely, amoeboid motility is characterised by squeezing movements that allow cancer cells to glide through matrix barriers, without the use of MMPs and integrin engagement. Amoeboid movement is characterised by a rounded morphology, high Rho kinase signalling to drive elevated levels of actomyosin contractility. MMP inhibitors have been tested clinically but failed to have effect on tumour metastasis [4], probably due to the plasticity of tumour cells and their ability to invade in an amoeboid manner in the absence of protease activity [5].

Previous data report that the epithelial mesenchymal transition (EMT) is promoted by the induction of a transcriptional programme that has been associated with the activation of several key transcriptions factors, including Snail (SNAI1), Slug (SNAI2), Twist and ZEB-1/2. This transcriptional programme ultimately leads to the disruption of adherens junctions, activation of polarized cell motility and increased degradation of ECM through secretion of MMPs [6-8]. In addition to EMT, a second type of motility shift has been described as essential in tumour progression, i.e., mesenchymal amoeboid transition (MAT) [9]. MAT can be induced in cancer cells by pharmacological inhibition of integrin function or MMP activity, by p53 or p27 deficiency [10,11], as well as through the activation or re-expression of EphA2 [5,12-14]. Although MAT confers a clear advantage to metastatic processes, very little is known about the molecular events that promote this motility shift [12,13,15].

Mammary epithelial cells undergoing EMT are endowed with stem-cell features, generating anchorage-independent mammospheres, soft agar colonies, and tumours [16]. Accordingly, we previously reported that the contact with cancer associated fibroblasts promotes EMT in the neighbouring prostate carcinoma cells, allowing them to acquire stem cell traits [17]. On the other hand, nothing is known to our knowledge, about a possible transcriptional regulation of amoeboid motility. However, also for MAT we have recently discovered a possible link with stemness. Indeed, in prostate cancer and glioblastoma, EphA2 expression, which induces an amoeboid motility, has been associated with achievement of stemness markers, increased clonogenic potential and tumour growth [15,18].

Melanoma cells are endowed with great plasticity in migration. Indeed, we have recently demonstrated that melanoma cells are able to shift between mesenchymal and amoeboid motility: melanoma cells move mesenchymally in response to pro-inflammatory cytokines, whereas after re-expression of embryonic EphA2 receptor, they achieve an amoeboid motility style giving rise to successful metastatisation [13]. Furthermore, Sanz-Moreno *et al*. showed that A375M2 primary melanoma cells can switch *ad hoc* between mesenchymal and amoeboid motility [19]. Furthermore, the same authors have recently demonstrated that treatment of melanoma cells with the Src inhibitor dasatinib results in a switch from mesenchymal migration to ROCK-dependent amoeboid invasion, confirming, once again, that cancer cell migratory capabilities could be blocked only by a combination of different treatments effective in the inhibition of both mesenchymal and amoeboid motility styles [20]. To confirm that cancer cells often undergo plasticity in cell motility, the opposite transition has been also described: the group of Marshall demonstrated that A375 M2 melanoma cells move in a rounded, amoeboid manner on top of or through collagen matrices due to JAK1-dependent MLC2 phosphorylation, whereas silencing of JAK1 induces a reduction in the acto-myosin contractility and the acquisition of an elongated morphology [21]. Moreover, the block of p53 function is sufficient to convert melanoma cells from an elongated motility style to a rounded locomotion, suggesting that such switch would favour the dissemination of p53-defective tumour cells by increasing their invasiveness [11].

In this light, the aim of our work is to investigate the regulation of mesenchymal to amoeboid transition induced in human melanoma cells by different stimuli and the possible link with the acquisition of clonogenic potential in order to sustain tumour growth in response to changes in microenvironmental conditions.

## Results and discussion

### EphA2 or RacN17 overexpression, treatment with Rho activator or ilomastat induces an amoeboid motility style in Hs294T melanoma cells

**Figure 1:**
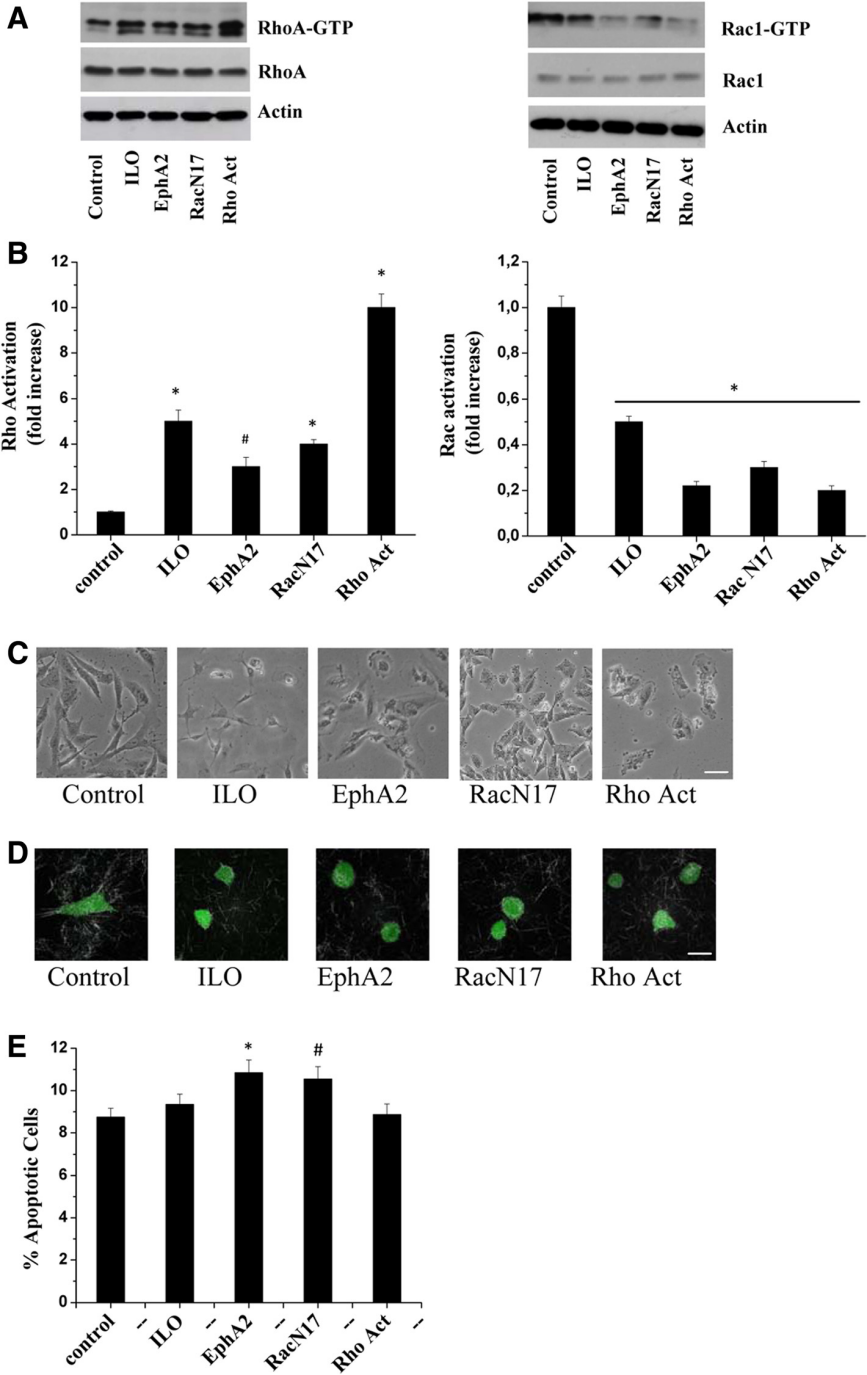
**EphA2 or RacN17 overexpression, treatment with Rho activator or ilomastat induce RhoA activation and Rac1 inhibition and acquisition of a rounded morphology. A)** 10^6^ Hs294T, EphA2 or RacN17 transfected cells were serum starved for 48 h, for Ilomastat treatment cells were serum starved for 48 h in the presence of 50 μmol/L Ilomastat, for Rho activation treatment cells were serum starved for 48 h and then stimulated with 1 U/ml Calpeptin for 2 h at 37°C. After treatments, RhoA-GTP and Rac1-GTP were analysed by pull-down assay from cell lysates. The total amount of RhoA and Rac1 were quantified by anti-Rho and anti-Rac1 immunoblot. An anti-actin antibody was used to ensure equal protein loading. The bar graphs obtained from densitometry analysis of triplicate experiments are shown. Student t-test, *p < 0.001 treatments *vs* control, #p < 0.005 treatments *vs* control. **B)** Cells were treated as in **A)**, photographs were taken and a representative image of their morphology is shown. Bar, 20 μm. **C)** Cell treated as in **A)** were labeled with CFSE and then incubated in three-dimensional collagen lattice. Cell morphology was monitored by confocal fluorescence-reflection microscopy. Photographs are representative of several randomly chosen fields. Bar, 20 μm. **D)** Cells were treated as in **A)**. The percentage of apoptotic cells was evaluated using the Muse™ Annexin V & Dead Cell kit. The results are representative of three experiments with similar results. Student t-test, *p < 0.01 EphA2 *vs* control, #p < 0.05 RacN17 *vs* control.

**Figure 2:**
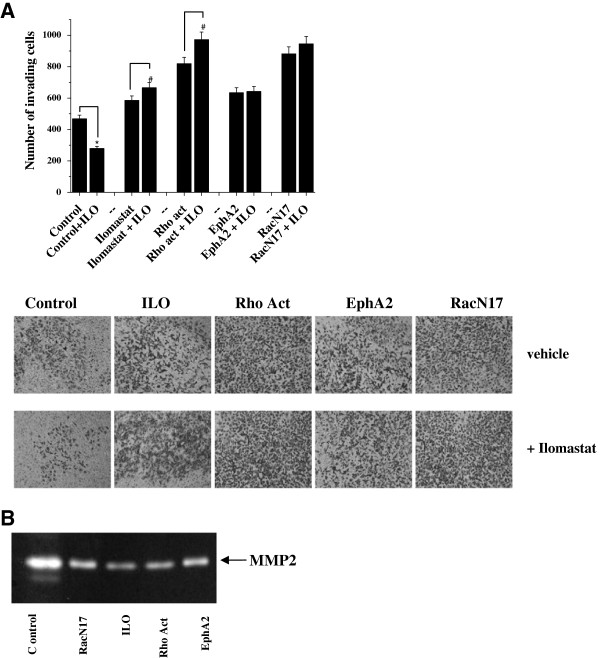
**Cells overexpressing EphA2 or RacN17, treated with Rho activator or ilomastat acquire an amoeboid- MMP-independent motility style. A)** Hs294T, EphA2 or RacN17 transfected cells were serum starved for 48 h, for Ilomastat treatment cells were serum starved for 48 h in the presence of 50 μmol/L Ilomastat, for Rho activation treatment cells were serum starved for 48 h and then stimulated with 1 U/ml Calpeptin for 2 h at 37°C. Then, 6×10^4^ cells were seeded into the upper compartment of Boyden chamber with or without 50 μM Ilomastat. Cells were allowed to migrate through the filter coated with Matrigel toward the lower compartment filled with complete medium. Cell invasion was evaluated after Diff-Quick staining by counting cells in six randomly chosen fields. The results are representative of three experiments with similar results. Student t-test, *p < 0.001 ILO treatment *vs* untreated, #p < 0.05 ILO treatment *vs* untreated. **B)** Analysis of MMP activity. Media from confluent monolayers of cells treated as in A) were collected and analysed by gelatine zymography. The clear bands represent areas of gelatinase activity. The results shown are representative of four experiments.

Previous studies from our laboratory demonstrated that overexpression of EphA2 in murine melanoma cells converts their migration style from mesenchymal to amoeboid like, thus conferring a cell plasticity in tumour invasiveness [13]. We now investigate the induction of an amoeboid motility style in human melanoma Hs294T cells following EphA2 overexpression and compare to amoeboid motility induced by RacN17 overexpression, treatment with the Rho activator Calpeptin or the MMPs inhibitor Ilomastat. We first analysed the activation level of RhoA and Rac1 small GTPases, as both RhoA activation and Rac1 inhibition have been correlated with a proteolysis independent motility style [12]. As shown in Figure 1A all these treatments are able to activate RhoA and to inhibit Rac1, thus suggesting a possible induction of an amoeboid motility in human melanoma cells. In addition, following all the aforementioned treatments, melanoma cells undergo cell rounding, a typical prerequisite for the acquisition of an amoeboid motility (Figure 1B). The confirmation that these cells undergo a real MAT emerges from the analysis of cell morphology in 3D collagen matrices, using confocal fluorescence reflection microscopy. As shown in Figure 1C all these treatments cause the acquisition of a round-shaped squeezing morphology while control cells maintain an elongated profile and establish contacts with collagen fibers. In addition, to exclude that the induction of the amoeboid morphology could be toxic for cells, we performed a cell viability assay. Figure 1D shows that none of the aforementioned treatments interfere with cell viability. To further investigate the motility of EphA2 or RacN17 overexpressing cells, as well as cells treated with Rho activator or Ilomastat, we tested their invasive abilities to cross a Matrigel barrier in the presence or absence of the MMPs inhibitor, Ilomastat. Indeed, sensitivity to protease inhibition of invasive ability of cells has been widely used as a mesenchymal/amoeboid discriminant test [13,15]. As shown in Figure 2A, control cells behaviour is highly influenced by the presence of Ilomastat. Conversely, cells exposed to MAT inducing treatments are completely unaffected by the presence of Ilomastat, suggesting that they preferentially use a MMPs-independent motility (Figure 2A). Indeed, the acquisition of an amoeboid motility style has been confirmed by MMPs analysis by gelatine zymography, which actually reveals both a decrease in expression and activation of MMP2 in cells overexpressing EphA2, RacN17 or treated with Rho activator or Ilomastat (Figure 2B). Overall, these data confirm that Hs294T melanoma cells undergo a clear MAT in response to all treatments used, highlighting the great plasticity in cell motility of these tumour cells.

### EphA2 overexpression, treatment with Rho activator or ilomastat activate common signalling pathways to achieve amoeboid motility

It is now well established that EMT is an epigenetic programme, mainly regulated at a transcriptional level, involving several factors, such as Snail, Slug, Twist, Goosecoid, ZEB1, and SIP1 [22,23]. We next investigated whether MAT induction displays common transcriptional traits independently of the stimuli that activate the MAT programme by gene expression profiling on Hs294T cells overexpressing EphA2, or treated with Ilomastat or the Rho activator Calpeptin. A class comparison was performed between treated cells and controls, ranking all genes according to Student t-test statistics. Gene set enrichment analysis (GSEA) [24] was applied to such ranked list to identify gene sets directly or inversely associated with MAT inducing treatments (i.e. EphA2, Ilomastat and Rho activator). The GSEA analysis revealed that MAT induction, independently of the stimulus that has activated the MAT programme, associates with several biofunctions represented by multiple gene sets, as reported in Table 1 and Additional file 1: Table S1. Notably, GSEA analysis revealed that the activation of the MAT programme associates with the repression of features that are characteristics of cell undergoing EMT as shown by a negative correlation with ANASTOSIOU_CANCER_MESENCHYMAL_TRANSITION_SIGNATURE gene set. However, a positive correlation was found with gene sets that are related to TGF-β, a well-known EMT inducer, targets of the EMT activator ZEB1 and with targets that are down regulated by E-cadherin expression (Table 1, epithelial mesenchymal transition). This let us speculate that MAT programme is not just a phenomenon that recapitulates the mesenchymal-to-epithelial transition (MET). The ability to acquire an amoeboid motility confers the cancer cells characteristics that are both of mesenchymal and epithelial cells and therefore are features of aggressive cancer cells with high plasticity. In keeping with the fact that MAT is significantly regulated by RhoA activation [12], we observed a positive correlation with the BERENJENO_TRANSFORMED_BY_RHOA_UP gene sets (Table 1, Rho). Based on our data, we suggest a model where EMT and MAT are different status that a cancer cell can display during cancer progression. Particularly, the EMT has to be at least partially repressed to allow the cell to enter into the MAT status, suggesting a hierarchy between EMT and MAT where MAT is a consecutive event of the EMT programme. Crucially, this may explain why clinical trials aimed at blocking EMT using anti proteolytic agents did not succeed [4,5].

**Table 1:** GSEA analysis: gene sets significantly correlated with MAT-inducing treatments

**Biological category**	**Gene set**	**Size**	**NES**	**FDR (q-value)**	**Enriched in**
EGF signalling	NAGASHIMA_EGF_SIGNALING_UP	57	2.773	0.000	Treated
	NAGASHIMA_NRG1_SIGNALING_UP	169	2.826	0.000	Treated
	ZWANG_CLASS1_TRANSIENTLY_INDUCED_BY_EGF	497	2.455	0.000	Treated
	AMIT_EGF_RESPONSE_60_HELA	43	2.305	0.000	Treated
	ZWANG_CLASS3_TRANSIENTLY_INDUCED_BY_EGF	217	2.263	0.000	Treated
	AMIT_EGF_RESPONSE_240_HELA	59	2.177	0.001	Treated
	AMIT_EGF_RESPONSE_120_HELA	67	2.098	0.002	Treated
	AMIT_EGF_RESPONSE_480_MCF10A	42	2.034	0.004	Treated
	AMIT_EGF_RESPONSE_40_HELA	42	1.972	0.007	Treated
Epithelial-mesenchymal transition	ANASTASSIOU_CANCER_MESENCHYMAL_TRANSITION_SIGNATURE	63	-2.348	0.000	Control
	AIGNER_ZEB1_TARGETS	34	-2.148	0.002	Control
	ONDER_CDH1_TARGETS_1_DN	166	2.339	0.000	Treated
	KARLSSON_TGFB1_TARGETS_UP	124	2.426	0.000	Treated
	PLASARI_TGFB1_TARGETS_1HR_UP	34	2.296	0.000	Treated
Extracellular matrix	REACTOME_COLLAGEN_FORMATION	57	-2.547	0.000	Control
	PROTEINACEOUS_EXTRACELLULAR_MATRIX	95	-2.224	0.001	Control
	KEGG_ECM_RECEPTOR_INTERACTION	84	-2.264	0.001	Control
	PID_INTEGRIN1_PATHWAY	66	-2.200	0.001	Control
	EXTRACELLULAR_MATRIX_PART	54	-2.203	0.001	Control
	KEGG_CELL_ADHESION_MOLECULES_CAMS	130	-2.127	0.002	Control
	EXTRACELLULAR_MATRIX	96	-2.108	0.002	Control
	REACTOME_EXTRACELL_MATRIX_ORGANIZATION	83	-2.063	0.004	Control
	COLLAGEN	22	-1.994	0.007	Control
HOXA5 pathway	CHEN_HOXA5_TARGETS_9HR_UP	216	3.745	0.000	Treated
HDAC	SENESE_HDAC1_AND_HDAC2_TARGETS_UP	227	2.790	0.000	Treated
	SENESE_HDAC1_TARGETS_UP	429	2.968	0.000	Treated
	SENESE_HDAC2_TARGETS_UP	110	2.525	0.000	Treated
	SENESE_HDAC3_TARGETS_UP	471	2.281	0.000	Treated
Stemness	NGUYEN_NOTCH1_TARGETS_DN	85	2.258	0.000	Treated
	RAMALHO_STEMNESS_UP	201	2.089	0.002	Treated
Rho metabolic process	BERENJENO_TRANSFORMED_BY_RHOA_DN	381	-1.951	0.010	Control
	CELLULAR_PROTEIN_CATABOLIC_PROCESS	56	2.164	0.001	Treated
	REACTOME_DEADENYLATION_OF_MRNA	19	2.062	0.003	Treated
	PROTEIN_CATABOLIC_PROCESS	64	2.058	0.003	Treated
	MRNA_METABOLIC_PROCESS	83	1.940	0.009	Treated
	OXIDOREDUCTASE_ACTIVITY	281	-2.123	0.002	Control
	OXIDOREDUCTASE_ACTIVITY_GO_0016616	56	-1.991	0.007	Control
	OXIDOREDUCTASE_ACTIVITY_ACTING_ON_CH_OH_GROUP_OF_DONORS	62	-1.951	0.010	Control
Metastasis	CHANDRAN_METASTASIS_UP	197	1.975	0.007	Treated
	CHANDRAN_METASTASIS_TOP50_UP	37	1.967	0.007	Treated
	PEDERSEN_METASTASIS_BY_ERBB2_ISOFORM_1	45	2.300	0.000	Treated

It is well known that MAT is promoted in looser matrices and is independent on contacts between cells and ECM [25-27]. In agreement with this rearrangement of the ECM, many of the gene sets identified positively correlate with a decrease in the secretion of ECM components, e.g. collagen, and with a reduction of contacts between cellular receptors (Table 1, extracellular matrix gene sets). These data suggest that MAT transcriptional programme associates with a change of the matrix stiffness that supports amoeboid motility style. In fact, amoeboid moving cells that need to squeeze within ECM fibres do not rely on proteolytic degradation of robust ECM and would certainly benefit from the release of cell adhesion bindings, from loosen and relax ECM stiffness, as well as from changes in ECM composition.

Although we observed that EMT is impaired during MAT, some features of EMT, emerged also from GSEA analysis, are maintained in cells moving with amoeboid motility. Indeed, we observe that MAT inducers treatment of Hs294T cells positively associates with gene sets involved in *anoikis* resistance and cell survival such as Epidermal Growth Factor (EGF) and Neuregulin 1 (NRG1) (Table 1, EGF signalling). *Anoikis* resistance is an essential requirement for a cancer cell that leaves the primary site to survive in the blood stream in order to metastasize to distant sites. This ability is even more crucial for a cell that is moving with amoeboid motility, i.e. independently of integrin engagement [12]. A positive correlation of MAT inducers treatment of melanoma cells with EGF signalling in cells moving with amoeboid motility is in keeping with previous studies describing EGFR activation during protection from *anoikis *[28]. Indeed, cells can prevent *anoikis* through the oxidation/activation of the tyrosine kinase Src, thus granting the activation of pro-survival pathways through a Src-dependent and ligand-independent phosphorylation of EGFR, which leads to Bim degradation [28].

It is now well established that EMT correlates with the achievement of stemness traits in multiple cancer models [16,17]. In addition, we have recently demonstrated that in prostate carcinoma cells, EphA2 silencing induces the loss of amoeboid motility style as well as a decrease in stem cell markers, thus suggesting that also MAT can be related to stemness and tumour growth [15]. In keeping with these observations, we observed that MAT inducing treatments in melanoma cells positively correlate with stemness gene sets (Table 1, stemness), suggesting that the achievement of stemness traits is not limited to EMT programme, but is a more general feature associated with the plasticity of tumour cell motility. These data suggest that, although EMT is a transcriptional programme leading to achievement of stemness traits [16,17,29], the additional shift occurring in cancer cells undergoing MAT contributes and enhances these stem-like features, further promoting the spread of metastases. In keeping, GSEA analysis revealed that metastases associated gene sets positively correlates with MAT inducers treatment (Table 1, metastasis). Furthermore, following MAT induction in Hs294T cells, we observed (i) a positive correlation with gene sets related to protein catabolism and (ii) a negative correlation with anabolic processes (Table 1, metabolic process). The increase in catabolic processes, likely connected to autophagy and leading cancer cells to self-sustain their metabolism during starvation, is a very common feature of cancer cells. Indeed, several tumours are often exposed to oxygen or nutrient deprivation, owing to mass overgrowth and insufficient angiogenesis [30]. Engagement of self-cannibalism and autophagic strategies have been indicated as protective against environmental stress, nutrient deprivation or chemotherapy treatment [31-33]. Metabolic deregulation of cancer cells during tumour progression has now attracted the interest of oncologists and is now a new Hallmark of Cancer [34], but there are very few data describing the metabolic reprogramming of cancer cells upon changes in their motility styles to compare them with the output of our GSEA analysis. Interestingly, EMT has been correlated with enhancement of anabolic processes, increase in cell biomass and therefore in cancer growth. While MAT appears an exacerbation of EMT programme for several aspects (e.g. activation of survival pathways and achievement of stem-like traits), the metabolic features of EMT or MAT undergoing cells appear to diverge.

Similarly to what observed in EMT, we suppose that also for the MAT programme a typical transcriptional profile could be identified. MAT inducing treatments show a positive correlation with histone deacetylase (HDAC) related gene sets (Table 1, HDAC), a feature of chromatin rearrangement, thus suggesting that MAT inducing treatments impact on gene transcriptional regulation. Importantly, in all MAT inducing treatments we found an important positive correlation with the HOXA5 controlled pathways (Table 1, HOXA5). HOXA5 is a transcription factor with a crucial role during morphogenesis and tumourigenesis [35]. Although it has not yet been involved in MAT and studies on its role in control of motility are still at their infancy, HOXA5 has been implicated in repression of EMT through regulation of ZEB1 or Snail [36-38]. These indications are in keeping with our observation that MAT induces a repression of the mesenchymal phenotype (Table 1, epithelial-mesenchymal transition).

### MAT promotes an increase in stem cell markers, self-renewal of melanoma cells, tumour growth in nude mice

**Figure 3:**
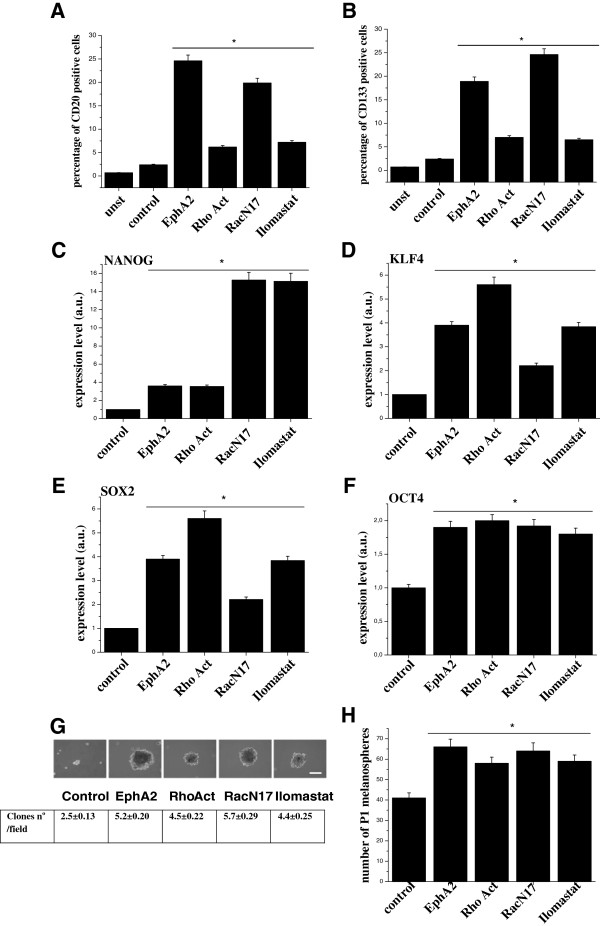
**Cells overexpressing EphA2 or RacN17, treated with Rho activator or ilomastat promote MAT and induce an increase in stem cell markers and clonogenic potential. A-B)** Hs294T, EphA2 or RacN17 transfected cells were serum starved for 48 h, for Ilomastat treatment cells were serum starved for 48 h in the presence of 50 μmol/L Ilomastat, for Rho activation treatment cells were serum starved for 48 h and then stimulated with 1 U/ml Calpeptin for 2 h at 37°C. Cells were then analysed for expression of the cell-surface markers CD20 and CD133 by means of cytometry. The CD20 or and CD133-positive populations were plotted. The results are representative of three experiments with similar results. Student t-test, *p < 0.001 treatments *vs* control. **C-F)** Cells were treated as in **A)**. Total RNA was extracted and NANOG, KLF4, SOX2 and OCT4 mRNA expression level was analyzed by qRT-PCR. Results are representative of three experiments with similar results. Student t-test, *p < 0.005 treatments vs control. **G)** Representative images of clones obtained from cells treated as in **A)** after 10 days of culturing at clonal densities. Bar, 100 μm. Clones were photographed, counted and the mean ± SD of clones number/field is reported. **H)** P1 individual spheres, derived from dissociated single melanospheres, were counted and the bar graphs obtained from triplicate experiments are shown. Student t-test, *p < 0.005.

To further investigate the link between stemness and MAT, we decided to analyse whether EphA2 or RacN17 overexpression, treatment with Rho activator or Ilomastat are able to further enhance the stemness of melanoma cells. Flow cytometry analysis of Hs294T cells reveals that all treatments inducing MAT enhance expression of CD20 and CD133, established stemness markers in melanoma (Figure 3A-B). In addition, qRT-PCR analysis showed increased levels of known embryonic stem cell factors like KLF4, NANOG, SOX2 and OCT4 which are involved in the maintenance of the undifferentiated state of stem cells and in the stem cell self-renewal [39,40] (Figure 3C-F). In keeping with the increase of the stem cell markers, activation of MAT increases the clonogenic potential of Hs294T cells, assessed by melanospheres formation assay and P1 melanospheres development (Figure 3G-H). The ability to form melanospheres is in keeping with anchorage independence and resistance to *anoikis* of Hs294T melanoma cells. We also confirm the link between MAT and stemness in a different cellular system, i.e. PC3 prostate carcinoma cells undergoing MAT in response to contact with endothelial cells [41]. Again, in MAT undergoing cells we observed an increase in stem cell markers, as well as an increase of the clonogenic potential (Additional file 2: Figure S1). These data confirm that MAT can induce a stem cell phenotype in different tumour types, independently of the MAT inducing stimuli.

**Figure 4:**
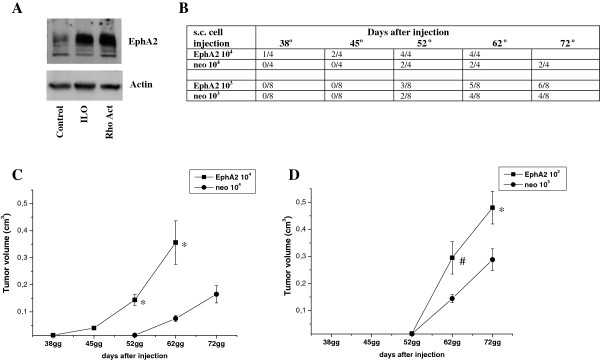
**The induction of MAT promotes tumor growth. A)** Analysis of EphA2 expression by immunoblot analysis of cells serum starved for 48 h with or without 50 μmol/L Ilomastat, or treated with 1 U/ml Calpeptin for 2 h at 37°C. An anti-actin antibody was used to ensure equal protein loading. **B)** Tumour incidence in SCID bg/bg mice injected with 10^4^ and 10^3^ EphA2 overexpressing cells or control cells. **C-D)** Xenograft growth in SCID bg/bg mice of EphA2 overexpressing cells or control cells s.c. injected with Matrigel in the flanks of mice. The onset and the primary tumour growth are reported. Student t-test, *p ≤ 0.001 EphA2 overexpressing cells *vs* control cells, #p < 0.005 EphA2 overexpressing cells *vs* control cells.

EphA2 expression is a common event during activation of MAT. In keeping with this, both Ilomastat and Rho activator induce EphA2 expression in melanoma cells (Figure 4A). For this reason, between the different treatments able to induce amoeboid motility, we selected EphA2 overexpressing cells to perform *in vivo* experiments. To test whether MAT could promote tumour growth *in vivo,* we compared the tumour initiating capacities of control melanoma cells and EphA2 overexpressing cells after s.c. injection into SCID-bg/bg mice. At lower concentration (10^3^ cells) EphA2 influences the rate of tumour growth and at higher concentration (10^4^ cells) both the onset and the growth of tumour are influenced by EphA2 overexpression (Figure 4C-D), thus demonstrating that the induction of MAT, in parallel with an enrichment of stem cell traits in Hs294T melanoma cells, drives an increase in tumourigenesis.

## Conclusion

In conclusion, MAT is likely to be an epigenetic invasive programme, hierarchically succeeding EMT, which further strengthens the stem-like and clonogenic features of cancer cells. For this reason, before fixing the concept that stemness is due to EMT engagement [16,17,23,29,36-38,42,43], it should be more correct to correlate stemness to enhanced plasticity in cells motility, a wider concept including EMT and MAT. Pharmacological strategies aimed at blocking only EMT are therefore destined to collide with the enormous adaptive and plastic features of cancer cells and should be revised by including MAT as an additional target of anti metastatic treatments.

## Methods

### Materials

Unless otherwise specified all reagents were obtained from Sigma. Anti EphA2 antibodies were from Upstate Biotechnology Inc. The invasion chambers were from Corning Costar. The Matrigel Matrix, anti Rac1 antibody were from RD System. Ilomastat was from Chemicon International. RNeasy Plus Mini kit was from Qiagen. Type I collagen, the FITC mouse anti human CD44 (clone G44-26) and PE mouse anti human CD24 (clone ML5) antibodies were from BD Bioscience (San Jose, CA, USA). CD133 and CD20 antibodies were from Abcam. Anti mouse Alexa 488 antibody was from Molecular Probes. The Rho activator was from Cytoskeleton. Magnetic Dynabeads CD31 for endothelial cell separation was obtained from Invitrogen.

### Cell culture and transfection

Hs294T human melanoma cells and prostate cancer cells (PC3) were purchased from ATCC and cultured in DMEM supplemented with 10% FCS at 37°C in 5% CO_2_ humidified atmosphere. Endothelial progenitor cells (EPCs) have been isolated from human umbilical cord blood as previously described [44,45]. EPCs were cultured on gelatin 1% coated dishes in EGM-2 medium (Lonza). Hs294T cells were transfected with RacN17 (a dominant negative mutant of Rac1) or EphA2 constructs using Lipofectamine 2000 (Invitrogen) according to manifacturer’s instructions.

### Analysis of cell morphology in 3D matrix

Cells were labeled by 5 μmol/L Cell Trace CFSE (Invitrogen) for 30 min at 37°C. Cells were then detached by Accutase (Sigma), washed and incorporated into 3-dimensional (3D) collagen I lattice (1.67 mg/mL rat tail collagen I). After 5 h, photographs were taken under confocal microscopy (Leica-SP5 system).

### Cell viability assay

10^5^ cells were detached using Accutase (Sigma) and suspended with 100 μl of the Muse™ Annexin V & Dead Cell Reagent (Millipore) according to manufacturer’s instructions. After 20 min, the percentage of apoptotic cells was analyzed by the Muse™ Cell Analyzer (Millipore).

### RhoA or Rac1 activity assay

Cells were directly lysed in RIPA buffer, the lysates were clarified by centrifugation and RhoA-GTP or Rac-GTP were quantified. Briefly, lysates were incubated with 10 μg Rhotekin-GST fusion protein (Becton Dickinson) or p21 activated kinase (PAK)-GST fusion protein, both absorbed on glutathione Sepharose beads for 1 h at 4°C. Immunoreactive RhoA or Rac1 were then quantified by western blot analysis. Lysates were normalised for RhoA or Rac1 content by immunoblot.

### Western blot analysis

1×10^6^ cells were lysed for 20 min on ice in 500 μl of complete radioimmunoprecipitation assay (RIPA) lysis buffer [50 mM Tris–HCl (pH 7.5), 150 mM NaCl, 1% NP40, 2 mM EGTA, 1 mM sodiumorthovanadate, 1 mM phenylmethylsulfonylfluoride, 10 μg/ml aprotinin, 10 μg/ml leupeptin]. Lysates were clarified by centrifuging, separated by SDS-PAGE, and transferred onto nitrocellulose. The immunoblots were incubated in 3% bovine serum albumin, 10 mM Tris–HCl (pH 7.5), 1 mM EDTA and 0.1% Tween 20 for 1 h at room temperature and were probed first with specific antibodies and then with secondary antibodies.

### Cell co-cultures

PC3 were cultured with EPCs (1:1 ratio) in EGM-2 serum-free medium for 48 h. PC3 cells alone were plated as a control. At the end of the co-culture, cells were separated using magnetic Dynabeads CD31 (Invitrogen) according to manufacturer’s instructions.

### Invasion assay

Cells were serum starved for 48 h and then 6×10^4^ cells were seeded onto Matrigel-precoated Boyden chamber (8 mm pore size, 6.5 mm diameter, 12.5 μg Matrigel/filter) with or without 50 μM Ilomastat. In the lower chamber, complete medium was added as chemo attractant. Following 24 h of incubation, the inserts were removed and the non invading cells on the upper surface were removed with a cotton swab. The filters were then stained using the Diff-Quick kit (BD Biosciences) and photographs of randomly chosen fields are taken.

### Gelatin zymography

Serum free medium from monolayer of cells was collected and 20 μl were added to sample buffer (SDS 0.4%, 2% glycerol, 10 mM Tris–HCl, pH 6.8, 0.001% bromphenol blue). The sample were run on a 10% SDS gel containing 0.1% gelatin. After electrophoresis the gel was washed twice with 2.5% Triton X-100 and once with reaction buffer (50 mM Tris–HCl, pH 7.5, 200 mM NaCl, 5 mM CaCl_2_). The gel was incubated over night at 37°C with freshly added reaction buffer and stained with Laemli Comassie blue solution. Areas of gelatinase activity appear as clear bands against a dark background.

### Gene expression profiling

Hs294T were serum starved for 48 h and in the presence of 50 μmol/L Ilomastat or serum starved for 48 h and treated with the Rho activator Calpeptin 1 U/ml for the last 2 h of incubation. EphA2-overexpressing Hs294T cells were serum starved for 48 h. Total RNA was isolated from Hs294T cells using RNeasy Plus Mini kit (Qiagen). Duplicate sample from 2 independent experiments were hybridized onto Human AffymetrixHuGene St 1.0 GeneChip array (Affymetrix) by Microarray Unit Cogentech (Milan, Italy). Data were normalised by RMA (Robust Multichip Analysis) algorithm using the *affy* package of Bioconductor/R. Microarray data have been uploaded in GEO (GSE52246). Class comparison between treated and controls samples was performed using a moderated t-test as implemented in the *limma* package of Bioconductor/R. Gene set enrichment analysis was performed using the GSEA v2.0 [24] on the pre-ranked gene list by applying the t-test statistics as ranking criteria. Both C2 (canonical pathways and signatures from the literature) and C5 (gene ontology terms) gene set collections from the MSigDB database (http://www.broadinstitute.org/gsea/msigdb) were tested for enrichment and gene sets with FDR < 1% were considered significantly enriched. Several biofunctions, each supported by multiple enriched gene sets were identified and reported in Table 1 and a complete list of the gene sets is reported in Additional file 1: Table S1.

### Flow cytometer analysis

To determine the surface expression of CD20, CD133, CD44 and CD24 10^6^ cells were detached non-enzymatically with 2.5 mM EDTA and incubated with the antibodies according to manufacturer’s instructions in PBS containing 1% BSA for 1 h at 4°C. After washing with PBS/1% BSA cells were incubated with Alexa 488 labelled anti mouse antibodies for 30 min at 4°C. Upon washing, a flow cytometer analysis was performed.

### Real time RT-PCR

Total RNA from Hs294T melanoma cells was extracted using RNeasy (Qiagen) according to the manufacturer instructions. Strands of cDNA were synthesized using a high capacity cDNA reverse transcription kit (Applied Biosystem) using 1 μg of total RNA. For quantification of mRNA expression, Real-Time PCR, using Power SYBR green dye (Applied Biosystem) was done on a 7500 Fast Real Time PCR system (Applied Biosystem). The primers were NANOG: 5′-ACCTTGGCTGCCGTCTCTGG-3′ (forward), 5′-AGCAAAGCCTCCCAATCCCAAACA-3′ (reverse); KLF4: 5′-GCAGCCACCTGGCGAGTCTG-3′ (forward), 5′-CCGCCAGCGGTTATTCGGGG-3′ (reverse); SOX2 5′-GAGCTTTGCAGGAAGTTTGC-3′ (forward), 5′-GCAAGAAGCCTCTCCTTGAA-3′ (reverse); OCT4: 5′-TTTTGGTACCCCAGGCTATG-3′ (forward), 5′-GCAGGCACCTCAGTTTGAAT-3′ (reverse). Data were normalized to those obtained with Glyceraldehyde-3-phosphate deydrogenase primers. Results (mean ± SD) are the mean of three different experiments.

### Prostaspheres/melanospheres formation and clonogenicity assay

Cells were detached using Accutase (Sigma). For prostaspheres and melanospheres formation, single cells were plated at 150 cells/cm^2^ on low attachment 100 mm plate (Corning) in DMEM/F12 (Invitrogen, Carlsbad, CA, USA) supplemented with B27 and N2 (Invitrogen), 5 μg/ml insulin, 20 ng/ml bFGF and 20 ng/ml EGF for prostaspheres or supplemented with N2, 0.6% glucose, 20 μg/ml insulin, 10 ng/ml bFGF and 100 ng/ml EGF for melanospheres. Cells were grown under these conditions for 10 days and then prostaspheres and melanospheres were photographed. For the evaluation of self-renewal, a single melanosphere was dissociated in single cells with Accutase, and a diluition of one cell per well into 96-well low attachment plates was performed in order to isolate individual P1 melanospheres. Single-cell cloning was confirmed by microscopic analysis, and single clones were counted.

### *In vivo* experiments

Xenograft experiments were performed in agreement with national guidelines and approved by the ethical committee of Animal Welfare Office of Italian Work Ministry and conform to the legal mandates and Italian guidelines for the care and maintenance of laboratory animals. 6–8 weeks old male SCID-bg/bg mice (Charles River Laboratories International, Inc., Wilmington, MA, USA) were injected subcutaneously (s.c.), both in the right and left lateral flanks, with cells mixed in a *1:1* volume ratio with Matrigel*,* in a final volume of 200 μl. Animals were monitored, tumour size was measured by a caliper and tumour volumes determined by the length (*L*) and the width (*W*): *V* = (*LW*^2^)/2.

## Abbreviations

ECM: Extra cellular matrix; EMT: Epithelial mesenchymal transition; MAT: Mesenchymal epithelial transition; MMPs: Matrix metallo proteinases.

## Competing interests

The authors declare no competing interests.

## Authors’ contributions

MLT, EG, LI, AM, MR performed the experiments; MC, PG analysed microarray data; MLT, AM and PC wrote the manuscript. All authors read and approved the final manuscript.

## Supplementary Material

Additional file 1: Table S1 GSEA analysis output. Gene Set Enrichment Analysis was performed with C2 and C5 collections of gene sets as described in the Methods section. Click here for file

Additional file 2: Figure S1 MAT promotes stemness in prostate cancer cells. A) PC3 cells were co-cultured or not with EPC for 48 h in order to induce MAT, then photographs were taken, the arrow shows a representative cell that has acquired a rounded morphology. Bar, 50 μm. B) PC3 cells were treated as in A), after separation, PC3 cells were analysed for expression of the cell-surface marker FITC-CD44 and PE-CD24 and CD133 by means of cytometry. The CD44^high^/CD24^low^or CD133-positive populations were plotted. Results shown are representative of three experiments. Student t-test, *p < 0.001 PC3 in co-culture *vs* PC3 alone. C) Representative images of clones obtained from PC3 cells or PC3 co-cultured with EPC after 20 days of culturing at clonal densities. Bar, 100 μm. Click here for file
