# Supplementary figures and images for: Mesenchymal to amoeboid transition is associated with stem-like features of melanoma cells
Source: Cell Commun Signal. 2014 Apr 1;12:24. doi: 10.1186/1478-811X-12-24 (PMC4022383; doi:10.1186/1478-811X-12-24)

**A**

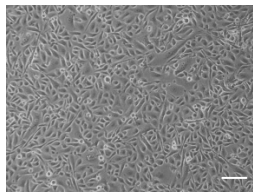

**PC3 alone**

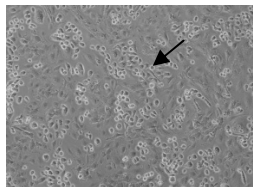

**PC3/EPC co-culture**

**C**

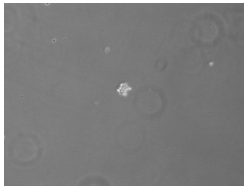

**PC3 alone**

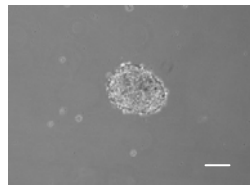

**PC3/EPC co-culture**

**B**

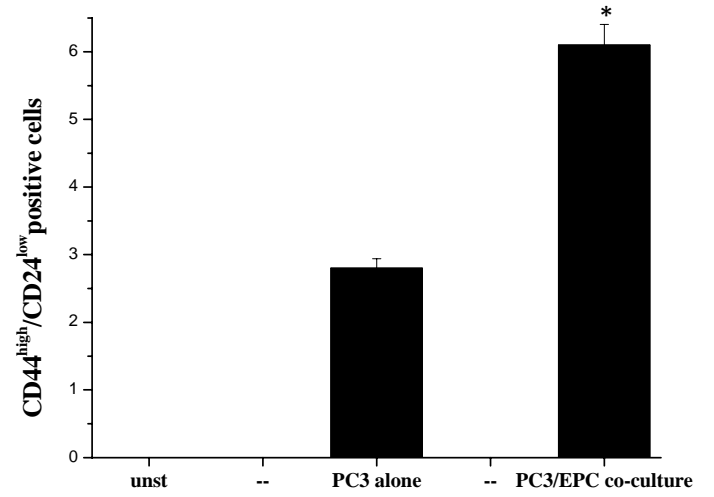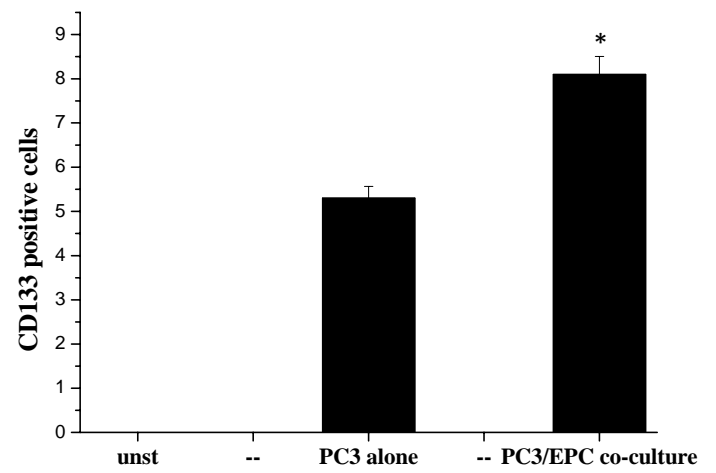

Supplement: Additional file 2: Figure S1 — MAT promotes stemness in prostate cancer cells. A) PC3 cells were co-cultured or not with EPC for 48 h in order to induce MAT, then photographs were taken, the arrow shows a representative cell that has acquired a rounded morphology. Bar, 50 μm. B) PC3 cells were treated as in A), after separation, PC3 cells were analysed for expression of the cell-surface marker FITC-CD44 and PE-CD24 and CD133 by means of cytometry. The CD44high/CD24lowor CD133-positive populations were plotted. Results shown are representative of three experiments. Student t-test, *p < 0.001 PC3 in co-culture vs PC3 alone. C) Representative images of clones obtained from PC3 cells or PC3 co-cultured with EPC after 20 days of culturing at clonal densities. Bar, 100 μm. [file 1478-811X-12-24-S2.pdf]
